# Intermunicipal travel networks of Mexico during the COVID-19 pandemic

**DOI:** 10.1038/s41598-023-35542-5

**Published:** 2023-05-26

**Authors:** Oscar Fontanelli, Plinio Guzmán, Amilcar Meneses-Viveros, Alfredo Hernández-Alvarez, Marisol Flores-Garrido, Gabriela Olmedo-Alvarez, Maribel Hernández-Rosales, Guillermo de Anda-Jáuregui

**Affiliations:** 1 CINVESTAV-IPN, Irapuato, Mexico; 2 Astronomer LTD, Cincinnati, USA; 3 Department of Computer Science, CINVESTAV-IPN, Mexico City, Mexico; 4 Centro de Ciencias Genómicas, UNAM, Cuernavaca, Mexico; 5 ENES Morelia, UNAM, Morelia, Mexico; 6 Computational Genomics Division, National Institute of Genomics Medicine, Mexico City, Mexico; 7 Investigadores e Investigadoras por México, National Council of Humanities, Sciences and Technologies, Mexico City, Mexico; 8 Centro de Ciencias de la Complejidad (C3), Universidad Nacional Autónoma de México, Mexico City, Mexico

**Keywords:** Complex networks, Socioeconomic scenarios

## Abstract

Human mobility networks are widely used for diverse studies in geography, sociology, and economics. In these networks, nodes usually represent places or regions and links refer to movement between them. They become essential when studying the spread of a virus, the planning of transit, or society’s local and global structures. Therefore, the construction and analysis of human mobility networks are crucial for a vast number of real-life applications. This work presents a collection of networks that describe the human travel patterns between municipalities in Mexico in the 2020–2021 period. Using anonymized mobile location data, we constructed directed, weighted networks representing the volume of travels between municipalities. We analysed changes in global, local, and mesoscale network features. We observe that changes in these features are associated with factors such as COVID-19 restrictions and population size. In general, the implementation of restrictions at the start of the COVID-19 pandemic in early 2020, induced more intense changes in network features than later events, which had a less notable impact in network features. These networks will result very useful for researchers and decision-makers in the areas of transportation, infrastructure planning, epidemic control and network science at large.

## Introduction

Intermunicipal mobility is a type of medium and large scale human mobility within a country where millions of individuals travel daily from one county or municipality to another, either going from home to work, shopping, accessing public services, cargo loading, vacation, etc. These movements and travels generate complex structures and dynamics of socio-economic interactions between different areas both at regional and national levels.

Given the nature of mobility systems, complex networks have been widely adopted to model commuting phenomena^1–4^^5^. Characterizing and understanding the properties of mobility networks is crucial for decision-making, urban planning, traffic engineering, and, as has become clear with the COVID-19 pandemic, designing, implementing and evaluating mobility restrictions and lockdowns to contain or control the epidemic spread^6–12^. Therefore, there is a need for public mobility network datasets aimed at researchers and decision-makers in the areas of geography, urban planning, epidemic control, etc. Traditionally these networks had been obtained from mobility surveys. However, the emergence of cell phone and GPS data in recent years has facilitated the acquisitions of accurate and large sets of human mobility data, thus allowing the construction and characterization of large and detailed human mobility networks.

The construction of origin-destination networks aiming to describe mobility patterns, measure the volume of public transportation and to plan public transportation has already been assessed. These mobility patterns include large-scale and long-range commuting patterns^13,14^, spatio-temporal patterns for different socioeconomic strata^4^, patterns in bike-sharing systems^15^, etc. Origin-destination matrices have also been used to measure the volume of use of public transport^16^^17,18^ and public transport planning^19,20^. Some approaches for modeling and generating origin-destination matrices include gravity models^21–23^, Bayesian models^24^ ,^25,26^, linear assignment matrix approximation^27^, Principal Components Analysis^28^ and gradient approximation method^29^, among others.

Origin-destination networks have been elaborated from different types of data. Most reports use data from the different public transport systems or road side interviews^21^. Alsger et al.^16^ and Munizaga and Palma^18^ used smart card fare data to estimate origin-destination networks. Lotero et al.^4^ considered data from bike-shared systems for these matrices. Wang and Mirchandani^17^ conducted experiments on two datasets generated by ride-hailing applications. Toledo and Kolechkina^27^ estimated the matrices using data from traffic counts on the network links. Recently, other works have attempted to utilize social network data from Twitter or Facebook^30–32^ ,^33^^34,35^ or data set from mobile phone data such ACAPS dataset or SafeGraph^36^ ,^37,38^. In particular, Edsberg et al.^34^ show that origin-destination networks based on data from cell phones and social networks present high-quality results.

Reconstructing mobility patterns is important in order to understand and model the spread of infectious diseases^39^. By analyzing the movements of individuals and populations, researchers can gain insights into how diseases may spread from one location to another. This information can be used to inform public health interventions and policies aimed at preventing the spread of disease. In particular, during the COVID-19 pandemic, the use of mobility data has been especially promising for incorporating realistic mobility flows into epidemiological models, helping to predict hospital admissions, and to assess the impact of mitigation policies on collective behavior. Additionally, the data can help refine interventions by providing near real-time information about changes in patterns of human movement, which can inform policy and messaging around social distancing and other interventions. Specific applications using many of the aforementioned data sources, from social networks^40^ to cellphone location data^41^.

Human mobility exhibits different behaviours across scales. At small spatial scales, such as within buildings or neighborhoods, people tend to move more randomly and frequently. At larger scales, such as between cities or countries, mobility is more likely to be directed and purposeful, with longer travel times and distances. These differences in scale are important for understanding various aspects of human behavior, such as socio-economic interactions and cultural dynamics. Recent work^42^ suggests that while human mobility may appear scale-free when looking at the overall distribution of displacements, there are meaningful scales corresponding to spatial containers that restrict mobility behavior. As such, specific approaches are needed to understand different scales of human mobility through empirical data.

Here we introduce a new public dataset of daily intermunicipal origin-destination networks in Mexico for 2020 and 2021, directly constructed from large datasets of geolocation data. This dataset will contribute to research and decision-making communities from diverse interests, from pure network theory to those studying human mobility, urban planning, national-scale social and economic relations, epidemic control.

This article is organized as follows: in the Results section, we present and describe the collection of mobility networks and show an analysis of changes in global (sum of weights), local (centrality measures), and mesoscale (community structure) network features. In Discussion, we argue how when initial COVID-19 restrictions were implemented in early 2020, more intense changes in network features were induced than in later events, which had a less notable impact on network features. In Methods, we describe the methodology we utilized to collect data and show the algorithm for network construction.

## Results

### A travel network dataset

We release a public dataset of 731 intermunicipal origin-destination networks in Mexico. These networks were constructed from a large and anonymized mobile location dataset (see^43^ for more information about this dataset). Each network is the intermunicipal origin-destination network in Mexico for each day during the 2020–2021 period. Nodes represent municipalities (third-level administrative division) or official metropolitan zones (see Methods section below). These are weighted and directed networks, where the weight of edge (*i*, *j*) is equal to the total number of observed travels from node *i* to node *j* normalized by the different number of mobile devices we recorded on that day. The data set with these 731 networks are freely available in an OSF repository http://dx.doi.org/10.17605/OSF.IO/42XQZ.

**Figure 1:**
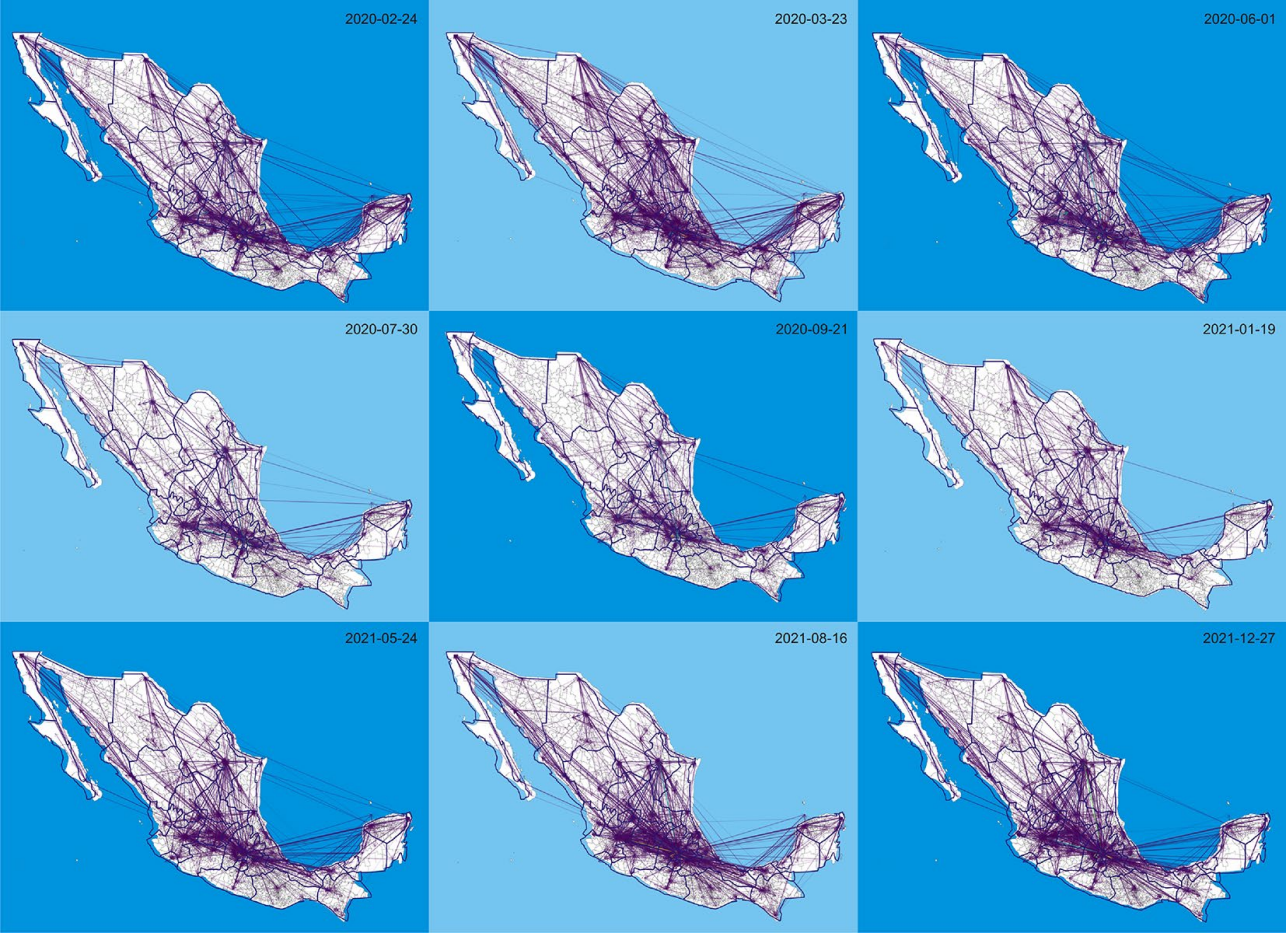
Network visualizations for selected days. On each network we show the 1$\%$ of edges with the highest weight.

For analysis and visualization purposes, we chose nine representative dates capturing different important events during the evolution of the pandemic in Mexico; these are shown in Table 1. As a first visualization, we show in Fig. 1 mobility networks over the Mexico map for this set of dates, drawing only the 1$\%$ of edges with the highest weight.

**Table 1:** Set of dates for the analysis. All of these make reference to events in Mexico.

Date	Event
2020-02-24	First reported COVID-19 case.
2020-03-23	Beginning of official lockdowns (National Program of Social Distance).
2020-06-01	Beginning of Epidemiological Stoplight Program.
2020-07-30	First national peak of daily contagions.
2020-09-21	Local minimum of daily contagions (between first and second wave).
2021-01-19	Second national peak of daily contagions.
2021-05-24	Local minimum of daily contagions (between second and third wave).
2021-08-16	Third national peak of daily contagions
2021-12-27	Local minimum of daily contagions (just before fourth wave)

### Changes in global network movement were observed throughout the first 2 years of the pandemic

In order to quantify the total observed movement in each network of the collection, we consider the total sum of weights in the network $S_{G} = \sum w_i$, where *i* runs over all edges of the network. In our context, a higher value of $S_{G}$ can be understood as higher mobility between municipalities in the country, which, in turn, is associated with people’s decisions to move outside their locality.

**Figure 2:**
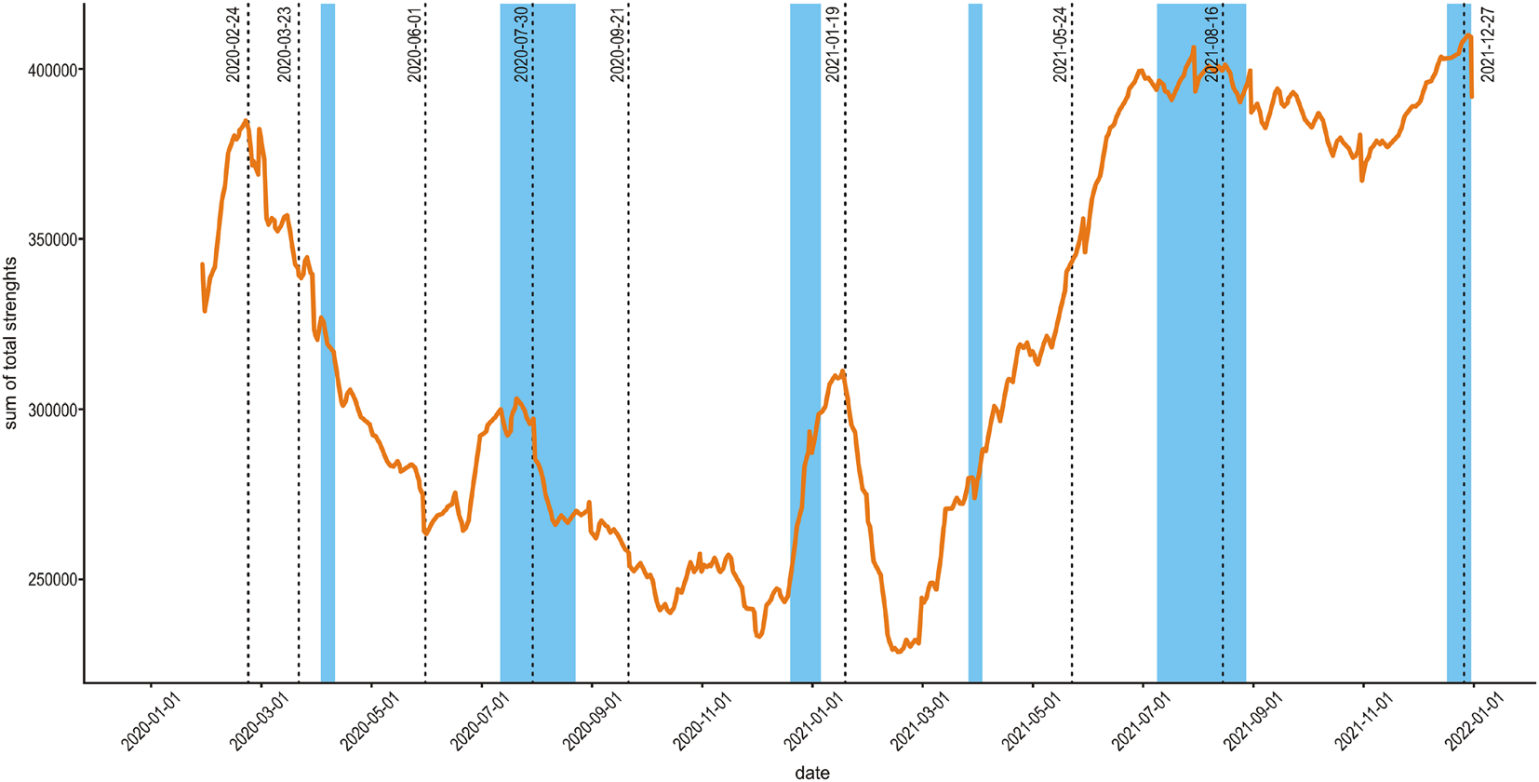
Time series of network edge weight sum with a 30 day moving average. The network edge weight sum is the sum of the weights of all edges in the network. These weights are a measure of the movement flow between two municipalities, as described in the method section. Vertical dotted lines correspond to dates shown in Table 1. Blue shaded areas are official school holiday periods (Easter holidays, from 2020-04-05 to 2020-04-14 and from 2021-03-28 to 2021-04-04; summer breaks, from 2020-07-11 to 2020-08-23 and from 2021-07-10 to 2021-08-29; winter breaks, from 2020-12-19 to 2021-01-05 and from 2021-12-18 to 2022-02-03.

Figure 2 shows the time series related to the $S_{G}$ metric, also indicating the set of dates described in Table 1 and official school vacation periods (summer breaks, winter breaks and Easter holidays, shaded in blue). The observed decay in mobility that starts in February 2020, is probably due to the post-holiday season, and is prolonged after the start of the lockdown, reaching a local minimum point shortly before the beginning of the summer holiday season. Following the summer break, mobility continued to decay, reaching its lowest point again shortly before the beginning of winter break, when there was a pronounced mobility rise. For the first half of 2021 we observe a sustained rise in mobility until July 2021, when it reaches a relatively high plateau.

### Node centrality measures highlight the dynamics of municipality movement

Since networks exhibit a different mobility pattern each day, centrality measures associated with each node (locality) also change. In this section we explore the variability of centrality measures over the period we studied. We choose three different centrality measures:

- *Degree centrality*: the number of inbound, outbound, or total adjacent edges to a given node. In this network, a single detected travel between two municipalities is enough to establish a link or edge between them.
- *Strength centrality*: the sum of the weights of inbound, outbound, or total adjacent edges to a given node. For an interpretation of the weight in these networks, see methods note 2.
- *Betweenness centrality*: a measure of the number of shortest paths that pass through a given node, indicating its role as a *bridge* between regions of a network.

For this section, variability is measured using the coefficient of variation (cv).

**Figure 3:**
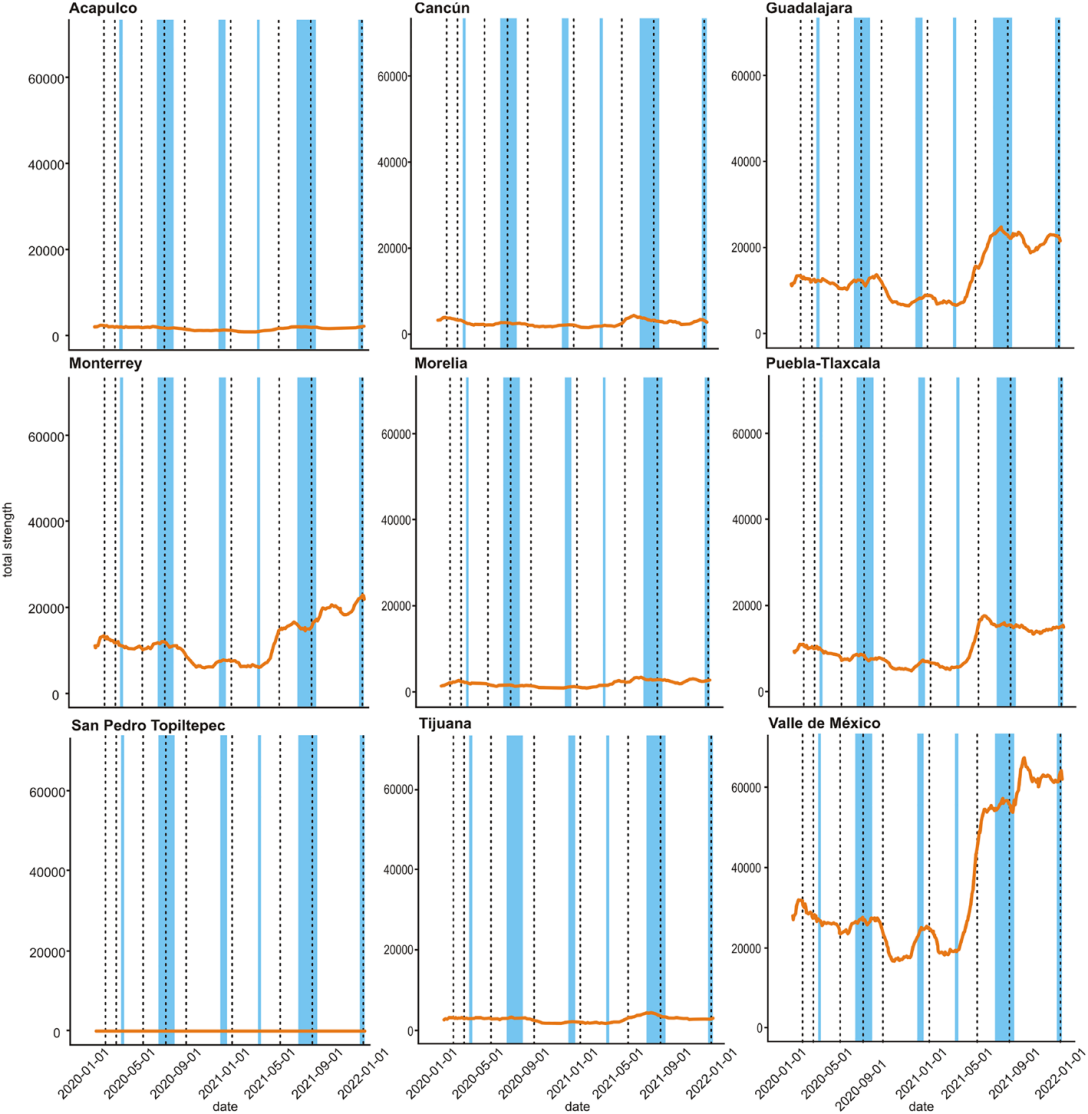
Time series of total strength for nine representative nodes. Node strength is defined as the sum of the weights of the edges adjacent to a given node; in this network, weights represent flows from and into the given node.

Figure 3 shows the variation of node strength for nine different nodes (municipalities or metropolitan zones). These nodes were chosen to illustrate different behaviours across the studied period. Figures corresponding to degree and betweenness centralities can be found in Supplementary Material Fig. 1 and Fig. 2.

The regions of Valle de México, Guadalajara, Monterrey and Puebla-Tlaxcala correspond to densely populated regions. In these areas, the coefficients of variation (100 $\times$ standard deviation/mean) for the three different centrality measures (degree, strength, and betweenness, respectively) are: (30.0, 53.1, 9.7) in Valle de México, (24.3, 50.3, 17.8) in Guadalajara, (31.3, 49.2, 25.9) in Monterrey, and (29.0, 44.0, 34.7) in Puebla-Tlaxcala. Morelia, a city in west-central Mexico, is included because it maintains a highly stable mobility pattern with coefficients of variation 24.0, 40.3 and 16.0 for the considered centrality measures. Meanwhile, places like San Pedro Topiltepec exhibit higher variability in their degree centrality; this is due to its having a mostly low strength throughout the analysis period, but exhibiting a higher strength during summer 2021; the coefficients of variation for this location are 320.4, 410.1 and 285.8. Tijuana is an important border city, so changes in centrality were expected whenever there were changes in the regulations on the border with the USA; the coefficients of variation are 34.9, 41.5 and 89.6. Finally, Acapulco and Cancún are cities that represent popular tourist beach destinations; the coefficients of variation for these cities are (41.4, 41.1, 60.2) and (33.3, 41.8, 62.1), respectively.

**Figure 4:**
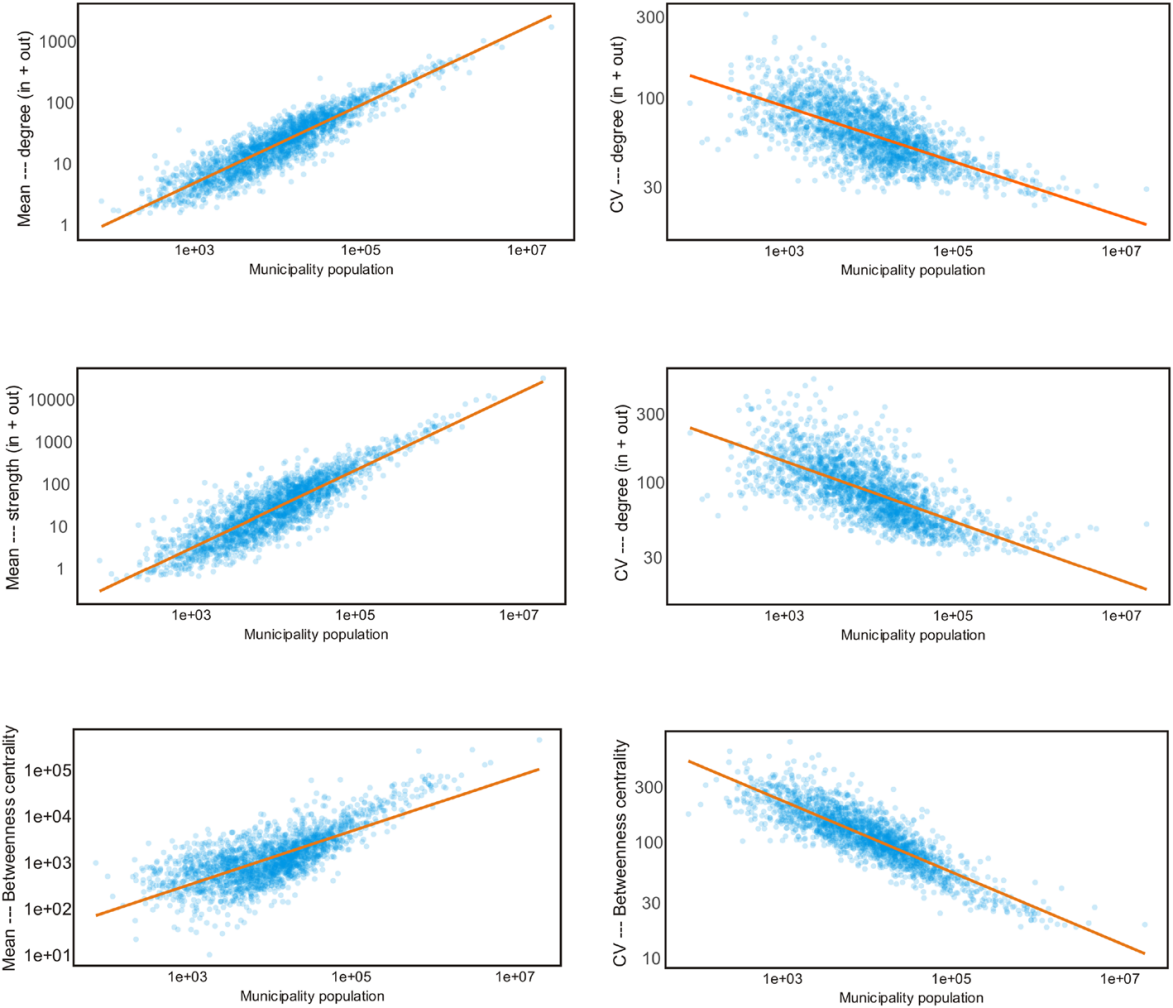
Left: log-log relationship between the mean of daily centrality measures and population (degree, strength, and betweenness centrality). Right: log-log relationship between the coefficient of variation of daily centrality measures and population (degree, strength, and betweenness centrality).

In order to explore the variability observed in node centrality measures with the population of the area they represent, the relationship between the three considered centrality measures and the population reported in the 2020 national census was analyzed as shown in Fig. 4 (census data taken from https://www.inegi.org.mx/programas/ccpv/2020/default.html#Datos_abiertos). Table 1 in Supplementary Material shows coefficients of determination when fitting linear regression models.

While the actual centrality values of each node may vary over time, it is important to notice that their overall ranking remains relatively constant; that is, regardless of the actual values at any given date, the ranking structure remains relatively stable. In Supplementary Material Fig. 3, we show the *rank turnover* time series; rank turnover is a recently proposed measure^44^ that quantifies the stability of a ranked list over time; we observe that the top 10, top 50, and top 100 nodes by either degree, strength or betweenness centrality are quite stable, compared to a null model of random daily rankings.

### Node strengths show asymmetric heavy-tailed distributions.

**Figure 5:**
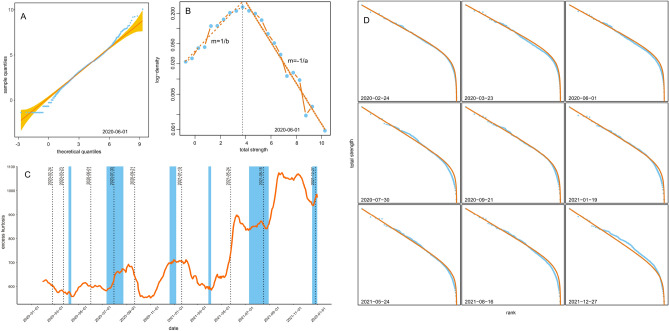
(**a**) Q-Q plot for total-strength distribution on 2020-06-01. (**b**) Histogram in semilog representation of total-strength logarithm on 2020-06-01, fails are fitted with parameters of Beta-Rank Function. (**c**) Time series for Kurtosis excess. (**d**) Rank-size plots and fits to Beta-Rank Function for a particular set of dates.

We performed several tests to study the distribution function of node strengths for the networks. Shapiro-Wilk test for the logarithm of node strength gives *p*-values below 0.01 for all 731 networks, where the largest *p*-value is equal to 0.004 for January 3rd, 2020. This value indicates that node strength distributions are not well fitted by a log-normal distribution. We show an example for a particular date in Fig. 5A, where we observe deviations from normality at both tails. Therefore, we calculated the excess kurtosis, which is a standard measure for tail extremity or the tendency to produce outliers^45^. We show in Fig. 5C a time series of excess kurtosis, where we observe that it takes positive values at all times.

It is worth noticing that the histogram of node strength in log-scale is not symmetric around the peak; an example of this can be seen in Fig. 5B. Consequently, we have applied the Beta Rank Function (BRF), which is a rank-size function, and a family of probability distributions that exhibits Paretian behavior in both tails, with different exponents^46^. BRF has the form of $X \sim (1-r)^b/r^a$, where *r* is the rank of the observation of size *X*, and *a*, *b* are free parameters. Unlike double-Pareto distribution, BRF has a smooth peak, and has been proved to be useful for fitting and modelling asymmetric heavy-tailed size distributions^47^. We can see how both tails decay from the peak with different rates, each one controlled by an independent parameter of the BRF. That BRF is a better model for our network node strength distribution is confirmed in rank-size plots shown in Fig.5D. Similar results hold for the rest of the networks. Analogous analysis for degree and betweenness distributions are shown in Supplementary Material Fig. 4 and Fig. 5.

### Formation and evolution of communities in the network.

Recalling that a community in a network is a set of nodes with a larger density of connections between them than external to the set, communities in these mobility networks correspond to groups of municipalities or metropolitan zones with a high internal mobility and a relative low external mobility (from the region to the outside or the other way around). Community detection algorithms in mobility or commuting networks have been widely used to detect or delimit geographical functional regions^48^^49^^50^^51^^52^.

**Figure 6:**
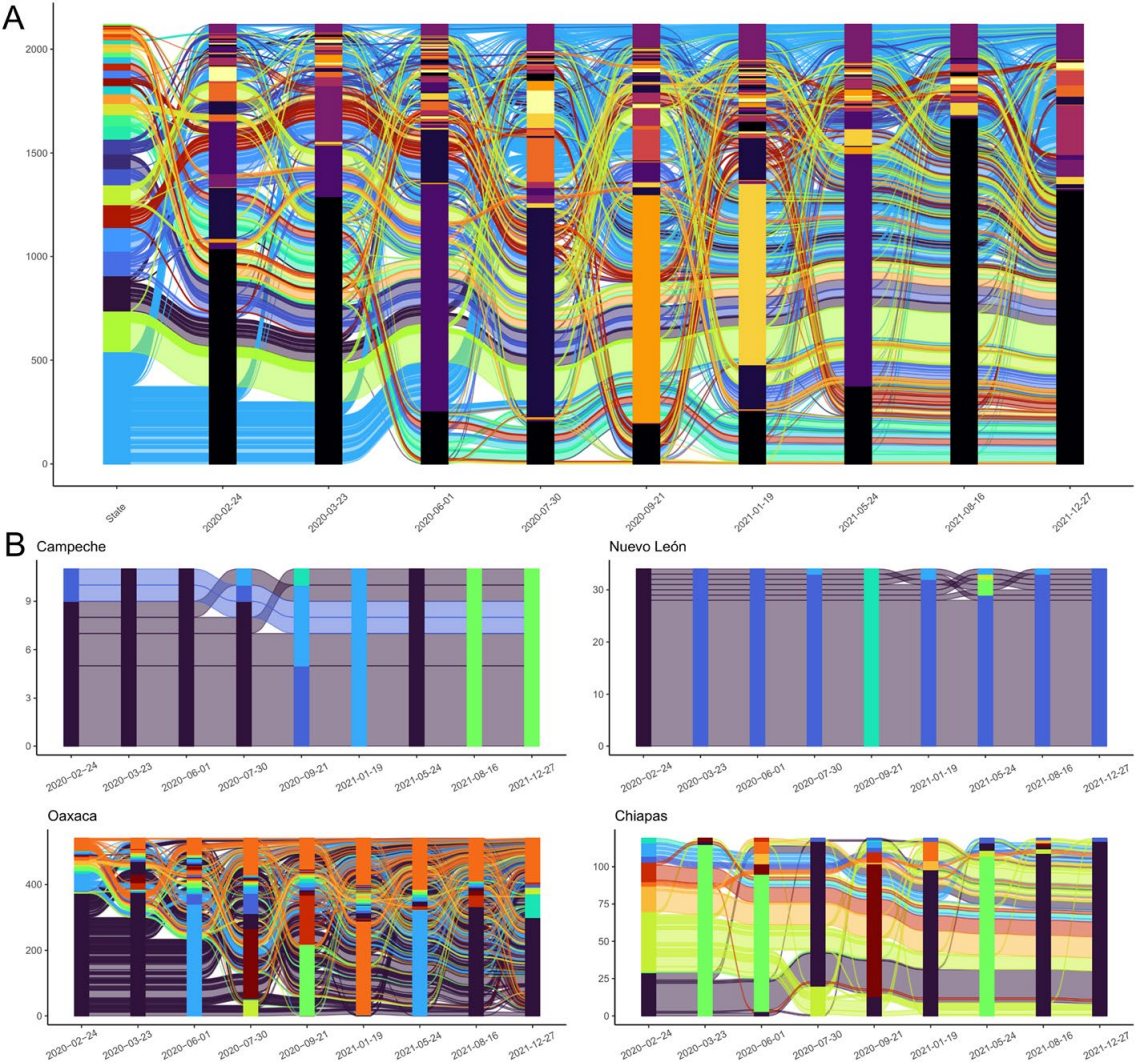
Panel (**A**): Alluvial plot for network communities for the set of nine relevant dates. Each thread represents a municipality or a metropolitan zone, coloured according to the state. For each day, nodes are grouped according to their community. For each day communities are shown in the vertical bars. Panel (**B**): alluvial plot for the subset of municipalities for four representative states, showing different community behaviours. In this visualization, line and bar color both represent community membership.

As a proof of concept, we have applied the label-propagation algorithm^53^ to detect communities in our networks. In Fig. 6, panel A, we show an alluvial plot that schematically shows the time evolution of community structure on the networks. In this diagram each line represents a municipality in Mexico grouped according to the network community they belong to. For the sake of visualization, the groupings shown correspond to the previously established representative dates. We observe that the number of communities increases during the lockdown period with respect to the pre-lockdown period; later, it shows a steep increase surrounding the start of the third COVID-19 wave - in summer 2021.

A question that may be asked in these networks is whether communities capture phenomena associated to geographic divisions; for instance, whether municipalities from the same state organize within single or many communities, and whether is common that these communities cross state lines. In Fig. 6, panel A, the color of each line represents the state to which that municipality belongs; we may observe that some communities are exclusively composed by municipalities of the same state, while others do cross state lines; in fact, we observe that interstate communities were more common just after the first national COVID-19 cases peak (in summer 2020, with about 50% of communities crossing state lines). Furthermore, we hypothesized that different states would have different community-forming behaviors; In Fig. 6, panel B, we show the alluvial plots for the subset of municipalities from four different states: Campeche, Nuevo León, Oaxaca, and Chiapas. Curiously, we observe that the first two states tend to have more stable communities that encompass a larger fraction of the state’s municipalities than Oaxaca and Chiapas.

At this stage, establishing a mechanism that describes why different municipalities behave differently in terms of community formation would be beyond the scope of this work; we should highlight, however, that we chose these four states as they are the two highest and lowest ranked states in terms of their GDP. Furthermore, the characterization of community structures in networks is not trivial, from the selection of the community detection algorithm forward; as such, we consider that the findings presented here serve mostly as a proof of concept of the type of analyses that this dataset may enable.

## Discussion

Analysis of global features, centrality measures and community formation in human mobility networks allow us to inquire about different kinds of effects that the pandemic may have had on intermunicipal mobility.

The sum of total weights over all edges of these mobility networks serves as an indicator of the total intermunicipal mobility that we observe on each day. We can observe in Fig. 2 a pre-pandemic peak at late February - early March 2020, since the first confirmed case in Mexico was reported on February 24, 2020. This peak is followed by a persistent decay and it continues to decrease from the previous maximum value observed in late February. In fact, this decay continues until June-July of 2020, when we observe a peak that coincides with the official summer break. After the summer break we still see a downward trend until the winter break and, again, we see a peak that coincides with an official vacation period. In late February 2021 we observe the lowest point of mobility and from here there is a sustained upward trend, apparently unaffected by Easter holidays until it reaches a new maximum that is even higher than the pre-pandemic peak. At this point mobility reaches a high plateau and, even though mobility still fluctuates, these fluctuations do not seem to be correlated neither with the pandemic third wave nor with vacation periods. From July 2021 total intermunicipal mobility stays at high levels, comparable with pre-pandemic levels. This is consistent with what we observe on the maps of Fig. 1; notice that, associated with the beginning of the traffic-light system and official lockdowns less mobility is visually observed (July and September 2020 show less mobility than March 2020) and how mobility increases on August 2021, coincident with the third peak of daily contagions.

Centrality measures evolve over time (Figs. 1 and 2). How they change seem to be weakly associated to the evolution of the pandemic. We also observe that not all nodes exhibit the same behavior. While some nodes, such as Guadalajara, Puebla-Tlaxcala and Valle de Mexico show larger fluctuations in their degree centrality, other nodes, such as Morelia exhibit more stable time series. Changes in betweenness centrality for the Valle de Mexico are not similar to those observed for other nodes. Evolution for degree and strength in Valle de Mexico seem to be correlated, but they differ from evolution of betweenness.

A relatively simple intuition is that municipalities with larger populations will have higher values of centrality measures regardless of time. We do see that all of the analysed centrality measures are correlated with population size. However, some of these metrics show a better linear correlation with population on linear scale while some other show a better linear correlation on logarithmic scale (see Table 1 in the Supplementary Material for model fits).

A related question is whether variations in these centrality measures are also correlated with population size. We observe a negative correlation between the coefficient of variation and population, showing that centrality variation is higher in smaller municipalities. Again, the best fit for this correlation is not necessarily observed in the linear scale. In any case, it should be noted that while population size is clearly a relevant factor, the dispersion of the point cloud in the range of midsized municipalities indicates other factors that may be in play to explain centrality measures in these networks fully.

Distributions of node strengths seem to be heavy tailed (Fig. 5). In light of Shapiro-Wilk results and the observation of the large values for the excess kurtosis, we can interpret this as a tendency to produce more outliers than the normal distribution. This means that a very few *hubs* in the network (nodes with very large strength) capture a very large portion of the total intermunicipal traffic. We observe a larger excess kurtosis during 2021 than during 2020 and a sustained upward trend with a few periods of very rapid increase. This translates into growing differences between large and small nodes in terms of strength. Regardless of how many people moved each year, this indicates that the way in which people travelled between municipalities exhibited changes through time. In addition to this, strength distributions are not symmetric around the peak, as a normal or log-normal distribution would be. This asymmetry - which deviates these distributions from a power law - suggests that these are not scale-free networks and that there exist at least two different regimes: one for large nodes and another for small nodes. Considering that these larger nodes have been identified to be larger metropolitan areas, whereas the smaller nodes tend to be smaller populations, this result may be related to the differential contribution of larger areas to intermunicipal mobility, whereas smaller towns contribute only to regional mobility.

Regarding formation of communities in the network, we could identify that for each day in this analysis there is a “giant” community that includes about half or more of the nodes in the network and that this structure is preserved for all dates. Therefore, if there are changes in the community structure of intermunicipal mobility, these have to occur at more local levels. In Fig. 6, each thread, which refers to a municipality in the network, is colored according to their state. For example, all light-blue threads starting at the bottom-left corner of the diagram correspond to Oaxaca municipalities, while green threads just above are Puebla municipalities. Notice here how all nodes in Puebla tend to stay inside the largest community, but municipalities in Oaxaca move to different communities, indicating a change in the structure of intermunicipal level mobility within this state.

When we wonder how communities form within different states we also see different behaviors (6B). Campeche and Nuevo León are two examples of states with a somewhat steady community structure, meaning that the majority of municipalities tend to stay in the same national community and that these community do not change much with time. In contrast, Oaxaca and Chiapas are states where municipalities fall in many different communities and they change from one community to another with time. This suggests the formation of local dynamics, where inhabitants move around small and specific groups of neighboring municipalities in a more considerable measure than people in Campeche and Nuevo León do.

It should be noted that the structure of this network is capturing a large-scale mobility behaviour. As previously mentioned, human mobility exhibits different characteristics at different scales^42^. In recent work, it has been shown that the granularity in which mobility is analyzed using digital data captures does capture some of the structural differences across scales^40^. Our research group’s work on studying these differences across scales is ongoing^43^.

It would be beneficial to have an external dataset for validating the mobility patterns observed in our study. In our previous work, we have already validated intrametropolitan mobility using official origin-destination networks^54^. Interestingly, the modular structure obtained using device mobility is similar to the one obtained from the official data. However, for intermunicipal travel, we were unable to obtain origin-destination data from the Mexican authorities, which hinders our ability to follow a similar validation strategy. Our future work aims to address this issue by exploring alternative data sources such as online social networks.

In summary, we present a set of daily intermunicipal origin-destination networks in Mexico for 2020 and 2021. These networks were constructed from geolocation information from mobile devices, using a large volume of data points. Making this dataset available to the community contributes in at least two important ways. First, it releases information that, by its nature and cost, can be difficult to access and does so with great detail and accuracy. Second, integrating location information into a network model eliminates the need to deal directly with point data and simplifies the task of exploring different dynamics between locations in Mexico.

Furthermore, the network collection was designed to include pandemic and pre-pandemic periods. This opens the possibility of using the set to explore changes in mobility patterns derived from the emergency, government regulations, and the decision of the general population to move between localities at different times during the health crisis.

To illustrate the potential use of network collection, we conducted several exploratory analysis focused on describing global and local changes in the networks and community detection. These tasks invite the formulation of questions that explore in-depth the latent patterns in the displacement dynamics captured by the networks.

We hope that making publicly the full dataset available will foster exchange among network scholars, be helpful to anyone interested in the dynamics that the country’s population creates between specific areas, and enrich the perspective on how such dynamics may be related to more complex processes.

## Methods

### Mobile device location data

We used mobile device location data for the time period between 2020-01-01 and 2021-12-31 within Mexican territory provided by Veraset, a company that aggregates anonymized mobile device location data. This source dataset is provided as a table in which each record (called a *ping*) contains the position (latitude and longitude) of a given (anonymized) device for a given timestamp (with temporal resolution up to seconds). The set of all unique device ids for a given day is called the *device panel*.

### Intermunicipal travel network construction

We define an Intermunicipal Travel Network (IMTN) as a directed, weighted graph *G*(*V*, *E*), for a given day, such that:

- Nodes represent localities (either municipalities or metropolitan zones, as defined by the national geographic agency; see Note 1)
- Links represent mobility from the source node to the target node, defined by observing at least one device that moved from node *i* to node *j*.
- Link weights represent the total fraction of observed devices that moved from node *i* to node *j*, out of the total number of observed devices; this acts as a normalized measure of flow between nodes (see Note 2).

### Complexity for network construction algorithm

Building intermunicipal travel networks requires large amounts of data and computation.The pseudocode in Algorithm 1 shows the algorithm used for network construction, starting with the daily mobile device location data. Complexity to compute these networks is $O\left( nm + l^2\right)$, where *n* is the number of pings, *m* is the number of municipalities and *l* is the number of devices. To build the networks, an average of 103,890,203 records per day were processed for 2 years and the average of devices per day was 3,239,070.

#### Note 1: on the aggregation of metropolitan area nodes

The political division of Mexico has municipalities as the smallest unit. Generally, a population center is contained within a municipality; however, there are large urban areas in which a single population center extends through many different municipalities, such that the movement between municipal boundaries is capturing the urban mobility and not travel between different locations. The National Geography and Statistics Institute (INEGI) defines 74 metropolitan areas in Mexico, based on measurements from 2015 https://www.inegi.org.mx/contenido/productos/prod_serv/contenidos/espanol/bvinegi/productos/nueva_estruc/702825006792.png.

Since intra-city mobility is beyond the scope of this manuscript, and would greatly skew the mobility metrics, as the volume of intra-city mobility is way larger than that of true travels between different locations, we decided to aggregate the municipalities that form these metropolitan areas into single nodes in the network.

#### Note 2: on the interpretation of edge weights in the network

We defined edge weights in the network as follows:

1 $$\begin{aligned} W_{ij} = \frac{|D_{ij}|}{|D|} \end{aligned}$$

where $|D_{ij}|$ is the number of devices that were observed to move from *i* to *j*, that is, were observed in *i* and their next immediate ping was in *j*; and |*D*| is the total number of devices in the day’s device panel. In this way, the weight represents a normalized measure of flow between regions; we may observe that in limit cases, the weight will be zero when there is no movement observed from one municipality to the other, and the weight would be 1 if all observed devices within the country travelled from region *i* to region *j*, which would be a virtually impossible scenario. An advantage of using this approach is that it controls variability in the number of observed devices each day, allowing for comparisons between days.
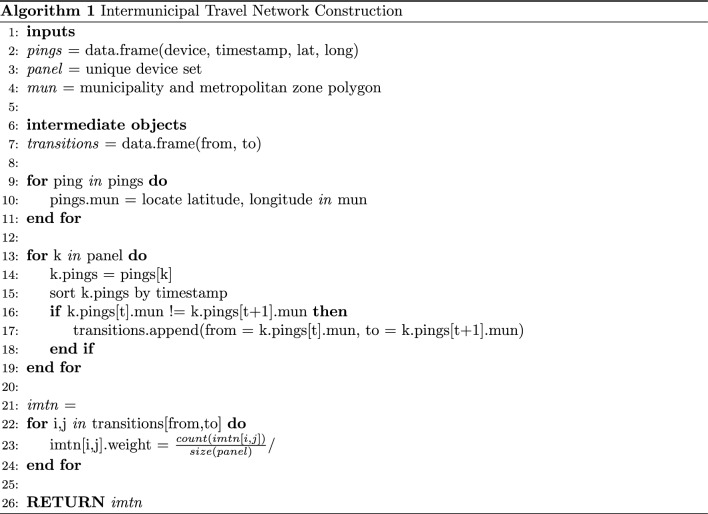


### Network analysis

Networks were analyzed using the igraph library, version 1.2.7^55^, for the R programming language, version 4.1.0. Rank turnover was calculated according to the definition found in^44^.

## Supplementary Information

Supplementary Information 1.

## Data Availability

The collection of 731 intermunicipal networks is publicly available on a OSF repository http://dx.doi.org/10.17605/OSF.IO/42XQZ.
