# Supplementary material for: Intermunicipal travel networks of Mexico during the COVID-19 pandemic
Source: Sci Rep. 2023 May 26;13:8566. doi: 10.1038/s41598-023-35542-5 (PMC10214328; doi:10.1038/s41598-023-35542-5)
Supplement: Supplementary file 1 — Supplementary Information 1. [file 41598_2023_35542_MOESM1_ESM.pdf]

Supplementary Material

In Figs. 1 and 2 we show time series for total degree and undirected betweenness centrality, respectively, for the nine representative nodes that we have picked. In these figures, we show in blue the dates of official school vacation periods en Mexico.

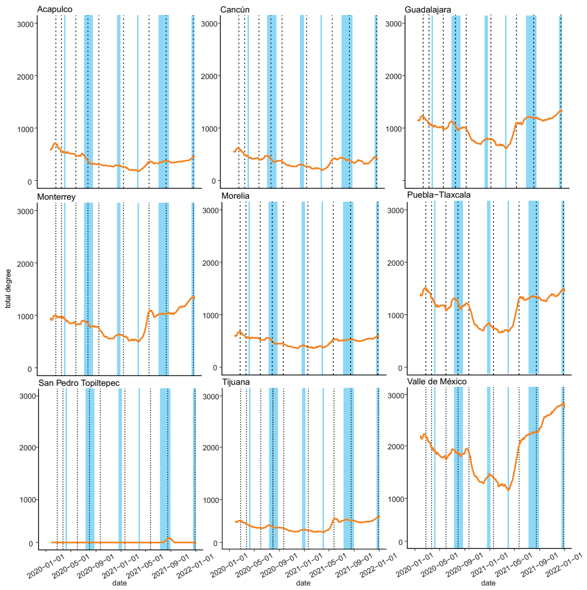

Supplementary Figure 1 : Time series of total degree for nine representative nodes representing municipalities or metropolitan areas.

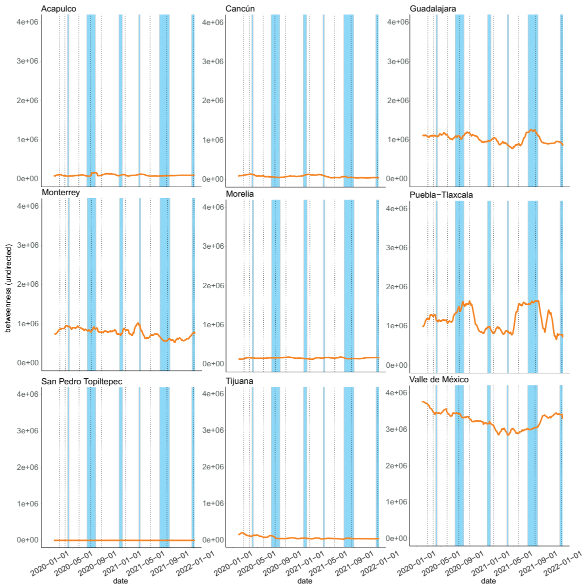

Supplementary Figure 2 : Time series of betweenness centrality for nine representative nodes representing municipalities or metropolitan areas.

In Table 1 we show the coefficient of determination (R squared) for the linear regressions we performed on node metrics against municipality population. We have two different kinds of regression models, one where the response variable is the temporal mean of the node metric and another where the response variable is the coefficient of variation (cv) of the node metric; in both cases the explanatory variable is municipality population. For some models we took logarithmic transformation on both the response and the explanatory variable, as we show on this table.

In Fig. 3 we show the results for the rank turnover of the highest ranked nodes by total degree, total strength and undirected betweenness. We compare these rank turnovers with a null model of randomly selected nodes. These results provide evidence that the empirical rankings in the mobility networks are

	Node metric	Statistic	Model	R squared
1	strength (in)	mean	lineal	0.94
2	strength (total)	mean	lineal	0.93
3	strength (out)	mean	lineal	0.93
4	degree (out)	mean	loglog	0.84
5	degree (total)	mean	loglog	0.84
6	degree (in)	mean	loglog	0.84
7	betweenness (undirected)	mean	lineal	0.74
8	betweenness (directed)	mean	lineal	0.69
9	betweenness (undirected)	cv	loglog	0.75
10	betweenness (directed)	cv	loglog	0.74
11	strength (out)	cv	loglog	0.50
12	degree (out)	cv	loglog	0.50
13	strength (in)	cv	loglog	0.50
14	strength (total)	cv	loglog	0.48
15	degree (in)	cv	loglog	0.48
16	degree (total)	cv	loglog	0.44

Supplementary Table 1 : Results for linear regressions for node metrics (mean and coefficient of variation) against population. Some of them were in lineal scale and some other in log scale.

more stable than what is expected by random turnover.

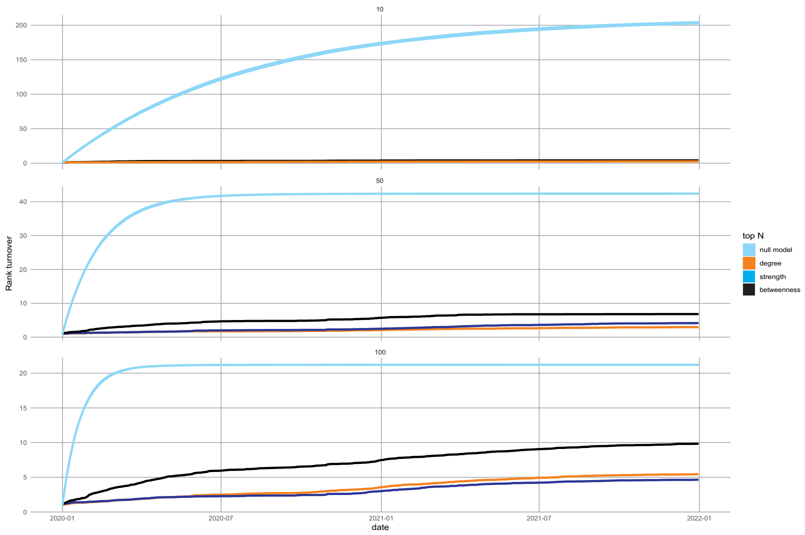

Supplementary Figure 3 : Rank turnover for top N highests ranked nodes by degree, strength, or betweenness centrality. Each panel shows the turnover (a measure of the number of new elements that join a top N ranked list across time) for N = 10, 50, or 100. In each panel, we show that the rank turnover increasing behavior for a null model, in which we randomly select a set of nodes at each iteration.

We show in Fig. 4 and Fig. 5 results for total degree distribution and undirected betweenness distribution, respectively. For degree distribution we observe that it has a heavy tail (positive excess kurtosis) and a histogram asymmetric with respect with the peak and a rank-size representation that deviates from power law. For node betweenness we observe a distribution with no regular form.

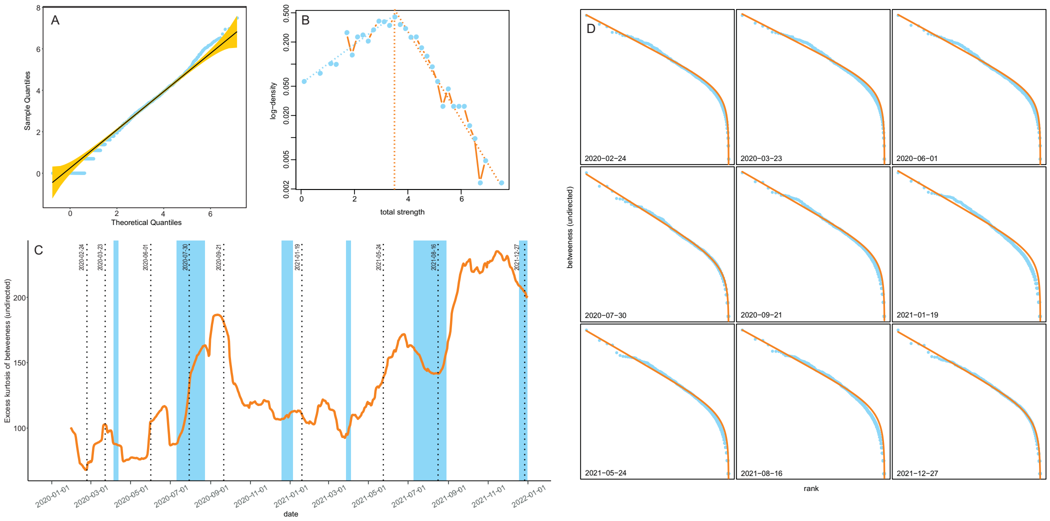

Supplementary Figure 4 : a) Q-Q plot for total-degree distribution on 2020-06-01. b) Histogram in semilog representation of total-degree logarithm on 2020-06-01, fails are fitted with parameters of Beta-Rank Function. c) Time series for excess-kurtosis. d) Rank-size plots and fits to Beta-Rank Function for a particular set of dates.

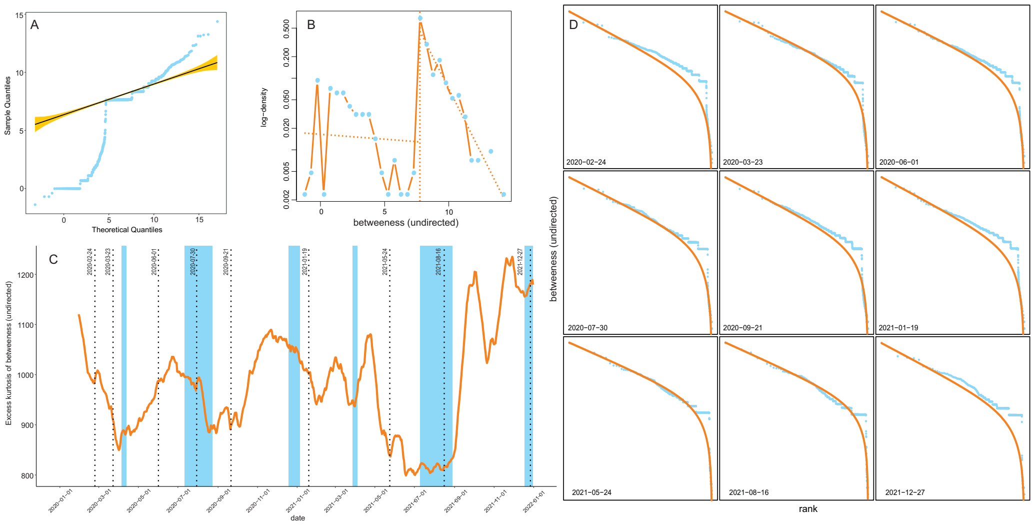

Supplementary Figure 5 : a) Q-Q plot for undirected betweenness distribution on 2020-06-01. b) Histogram in semilog representation of undifected betweenness logarithm on 2020-06-01, fails are fitted with parameters of BRF. c) Time series for excess-kurtosis. d) Rank-size plots and fits to BRF for a particular set of dates.
